# Self-Management Apps for People With Epilepsy: Systematic Analysis

**DOI:** 10.2196/22489

**Published:** 2021-05-28

**Authors:** Mohsen Zaied Alzamanan, Kheng-Seang Lim, Maizatul Akmar Ismail, Norjihan Abdul Ghani

**Affiliations:** 1 Department of Information Systems University of Malaya Kuala Lumpur Malaysia; 2 Division of Neurology, Department of Medicine, Faculty of Medicine University of Malaya Kuala Lumpur Malaysia; 3 Department of Information Systems Faculty of Computer Science and Information Technology University of Malaya Kuala Lumpur Malaysia

**Keywords:** mobile health, epilepsy, self-management, smartphone

## Abstract

**Background:**

Patients with epilepsy (PWEs) are motivated to manage and cope with their disorder themselves (ie, self-management [SM] is encouraged). Mobile health (mHealth) apps have multiple features that have a huge potential to improve SM of individuals with chronic disorders such as epilepsy.

**Objective:**

This study aimed to review all freely available apps related to the SM of PWEs and to determine the SM domains covered in these apps.

**Methods:**

We performed a search of apps on Google Play and App Store using the keywords “epilepsy” or “seizures” from May to August 2018. Apps were included if they were free and in English language. We excluded apps with installation-related issues and not related to epilepsy self-management (eSM).

**Results:**

A total of 22 eSM apps were identified in our search: 6 of these run only on iOS, 7 only on Android, and 9 run on both operating systems. Of the 11 domains of SM, seizure tracking and seizure response features were covered by most apps (n=22 and n=19, respectively), followed by treatment management (n=17) and medication adherence (n=15). Three apps (Epilepsy Journal, Epilepsy Tool Kit, and EpiDiary) were installed more than 10,000 times, with features focused specifically on a few domains (treatment management, medication adherence, health care communication, and seizure tracking). Two apps (Young Epilepsy and E-Epilepsy Inclusion) covered more than 6 SM domains but both had lower installation rates (5000+ and 100+, respectively).

**Conclusions:**

Both Android and iOS mHealth apps are available to improve SM in epilepsy, but the installation rate of most apps remains low. The SM features of these apps were different from one another, making it difficult to recommend a single app that completely fulfills the needs of PWEs. The common features of the apps evaluated included seizure tracking and seizure response. To improve the efficacy and availability of these apps, we propose the following: (1) involve the stakeholders, such as physicians, pharmacists, and PWEs, during the development of mHealth apps; (2) assess the efficacy and acceptance of the apps objectively by performing a usability analysis; and (3) promote the apps so that they benefit more PWEs.

## Introduction

Epilepsy affects approximately 50 million people of all ages worldwide, irrespective of race and economic background [1,2]. It is estimated that nearly 200,000 individuals living in Malaysia have epilepsy [3]. According to the World Health Organization, epilepsy is one of the most common neurological diseases [4]. Patients with epilepsy (PWEs) face many challenges related to their disease as well as to their families and health care providers (HCPs) [5]; additionally, lack of disease control affects the patients’ work, relationship, and daily responsibilities [6]. HCPs also find it hard to monitor their PWEs to understand if any of their symptoms are improving or if they are experiencing any new symptoms [7]. In Malaysia, based on our experience, most PWEs use the calendar for seizure monitoring and some also record their medications on it.

PWEs are encouraged to self-manage their condition, as this has been proven to improve quality of life and considers the priority of their care [8]. However, the features of existing apps vary from one another, making it difficult to recommend one that is most complete to fulfill the needs of PWEs.

Self-management (SM) is generally defined as an individual’s ability to manage the symptoms, treatment, physical and psychosocial consequences, and lifestyle modifications necessary when living with a chronic disease [9]. SM is a dynamic process, which includes steps and decisions taken daily in response to living with a chronic condition [9]. In this process, patients are empowered for changing their health behavior via better knowledge about their disease and treatment, and by managing symptoms and physical/psychosocial consequences of the disease [10]. Patients need to rely on their self to manage their disorder daily [11].

The Institute of Medicine advocates the development of epilepsy self-management (eSM) programs to ensure the patients benefit from technologies such as computers and mobile devices [12]. Mobile health (mHealth) apps, including eSM apps, present daily companions of health data which help patients and health care professionals improve health outcomes [13] through constant monitoring of the patient’s status.

mHealth apps play a major role among patients with chronic disease in managing their disorders [14]. According to one report, there were 325,000 mHealth apps on the market in 2017 [15]. There are about 165,000 mHealth apps in major app stores and most of these relate to management of diseases [16].

Many studies have found that mobile apps can assist patients with chronic condition [17]. Recently, a multitude of mHealth apps have been launched and shown to improve chronic disease care in patients with diabetes mellitus, hypertension, and other disorders [12,14,18].

The use of smartphone apps could be a promising strategy for seizure SM [14]. Nearly 94.4% of PWEs owned a smartphone; thus, it could be said that there is a positive attitude toward using epilepsy apps among PWEs [14].

This study aimed to perform a systematic review to identify the free eSM apps available in Apple App Store and Google Play app stores and to identify the domains covered by these eSM apps.

## Methods

### Study Overview

In this study we performed a systematic review of all apps that relate to PWEs and help these patients manage their disorder. We searched for eSM apps in both iOS (App Store) and Android (Google Play) app stores.

### Research Questions

This study aimed to answer the following 3 questions:

- Research Question 1: What are the apps available to help in eSM?
- Research Question 2: What are the features of these apps?
- Research Question 3: What is the user rating of mobile apps related to eSM?

### Inclusion Criteria

We performed a search of apps on Google Play and App Store using the keywords “epilepsy” or “seizures” from May to August 2018. The research was conducted in Malaysia; therefore, we included only Android and iOS apps, as only these are available in the country.

Inclusion criteria for choosing mobile apps were as follows: available in English, can be freely downloaded, and intended for individuals with epilepsy, covering at least one of the 11 domains described by Escoffery et al [9], which include health care communication, social support, medication adherence, treatment management, seizure response, wellness, stress management, coping and stress management, seizure tracking, safety, and proactivity.

### Exclusion Criteria

Apps with installation issues or in other languages were excluded.

### Review Process

To assess the quality of the apps and risk of bias, we first read and reviewed the app’s description on the download page, to ascertain that the app fulfilled the inclusion or exclusion criteria. However, the app description alone is not enough to assess the app’s quality [19], and therefore all selected apps were downloaded for further analysis [19]. A user’s rating scale (score range 0-5) was used to determine the quality of the apps, with 5 indicating the best quality.

## Results

### Search Strategy and App Characteristics

**Figure 1:**
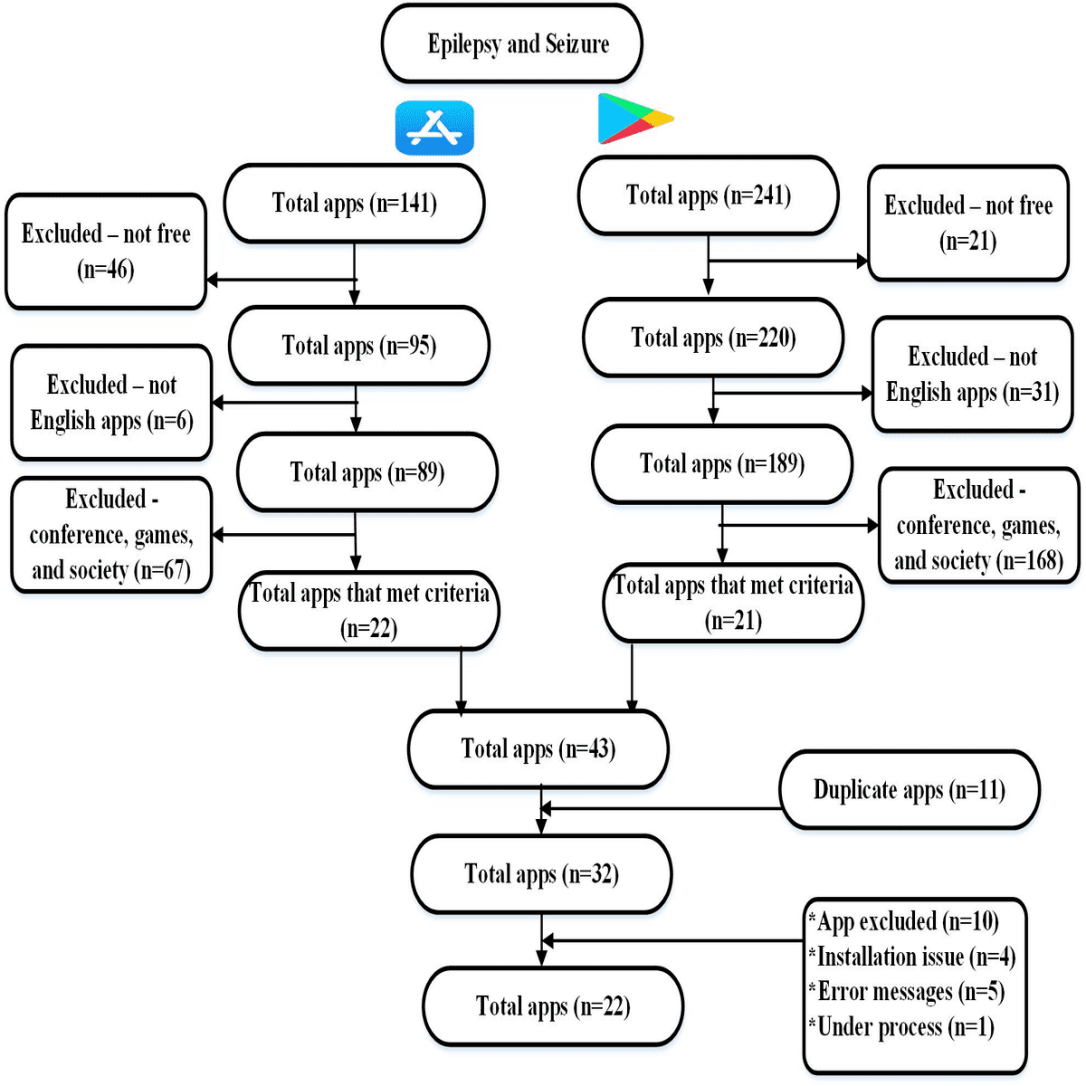
PRISMA flow diagram.

A total of 382 mobile apps were found in the 2 app stores: 241 for Android and 141 for iOS. Apps that were not in English and not free were excluded (n=104), thus eventually 278 apps were downloaded for analysis. Next, we reviewed the description of each app to determine its features and purposes and excluded 235 apps, such as conference (meeting), games, and flashlights, which were not related to eSM. Eventually, 43 apps fulfilled our criteria covering 1 or more eSM domains described by [9], of which 11 were compatible with both Android and iOS. We included 32 eSM apps for further analysis; however, 10 of these apps were excluded because of installation issues or they were malfunctioning. Table 1 presents the reasons for excluding the 10 apps during the installing stage. Of the final 22 included for analysis, 6 (27%) run on iOS, 7 (32%) on Android, and 9 (41%) on both operating systems. A PRISMA flow diagram showing the app selection process is presented in Figure 1. All 22 apps enhance eSM; however, none of the apps indicated whether any of the stakeholders, such as physicians, pharmacists, and PWEs, had participated in the app development process.

Most apps are generally complicated and the PWEs has to learn how to enter their data on the app, thereby affecting the potential use of these apps. Patients with chronic diseases such as epilepsy need a user-friendly app. As it is imperative that the content and features of epilepsy support apps motivate and support PWEs to manage their illness, PWEs and HCPs should be given an opportunity to participate and collaborate in developing a mobile app for eSM. The home page for each app contains a brief description about the app to convince the users to download it. This is especially the case with paid apps [19]. However, some app descriptions do not reflect the actual features and functions of the app. None of the apps indicated on their home page whether they have managed to achieve their goals through acceptance by the users. There was also no statement about the participation of stakeholders while developing the apps.

**Table 1:** Epilepsy self-management apps with issues during the installation stage.

App name	Operating system	Issue
Total Epilepsy Recorder	iOS	Error message
Seizure Sync Epilepsy Log	iOS	Error message
Fable Epilepsy Charity	iOS	App has been temporarily disabled by publisher
Wacean	iOS	Error message
Epilepsy Tracl	Android	This is currently a work in progress
Seizure Tracker	Android	Error message
Seizure Sync Epilepsy Manager	Android	Error message
Total Epilepsy	Android	Installation issue
Alert	Android	Installation issue
Dr. Epilepsy	Android	Installation issue

### User’s Rating

We used the apps’ home page to obtain the user ratings. Table 2 presents the average user rating for the apps.

The ratings shown in Table 2 also depict the popularity of certain apps, as rated by the users. An app is usually considered popular if the users are happy with it, which eventually provides an indication of its usefulness.

**Table 2:** App user rating.

Name (app developer)	Number of installations	Average user rating	Number of raters
Epilepsy Journal (Olly Tree Application)	10,000+	4.4	176
Epilepsy Tool Kit (Epilepsy Society)	10,000+	3.9	79
EpiDiary (EpiDiary Irody, Inc.)	10,000+	3.5	345
Young Epilepsy (Synergix Health Ltd.)	5000+	4.1	88
Seizure Log (Seizure Tracker LLC)	5000+	4.7	9
Simple Seizure (NA^a^)	5000+	4.4	176
My Seizure Diary (Epilepsy Foundation)	5000+	2.2	52
Epilepsy Ireland Diary App (DXC Technology)	1000+	3.1	11
Seizure First Aide (Afixia LLC)	1,000+	3.4	12
Win Over Epilepsy (WOE) (Bee Mobile Pvt. Ltd.)	500+	4.6	19
Birdhouse – for Epilepsy (Birdhouse LLC)	500+	3.0	2
EpApp (PENNSW & The Sydney Children’s Hospitals Network)	100+	3.8	8
BioMark Health Epilepsy (NA)	100+	4.7	12
E-Action Info (The ImageFactory)	100+	4.00	2
E-Epilepsy Inclusion (The Hong Kong Society for Rehabilitation)	100+	4.9	7
ElFY Epilepsy (ELFY APP)	10+	5.00	2
Helpilepsy – Epilepsy Assistant & Diary (M4KEIT)	500+	4.8	8
Epilepsy Help (Dr. Bindu Menon Foundations)	—^b^	4.8	16
Epilepsy Safe (Comalf)	—	3.7	6
Epi & Me 2 (HandMe)	—	0	—
M.E. (Epilepsy Foundation of New Jersey)	—	0	—
SAMi3 Sleep Activity Monitor (HiPass Design LIC)	—	—	0

^a^NA: not available.

^b^—: Not applicable

### Domains of Self-Management

Apps analyzed covered a total of 11 domains. The common domains were seizure tracking and seizure response, followed by treatment management and medication adherence. Three apps (Epilepsy Journal, Epilepsy Tool Kit, and EpiDiary) were installed more than 10,000 times, with their features specifically focusing on a few domains (treatment management, medication adherence, health care communication, and seizure tracking). Two apps covered over 6 SM domains (Young Epilepsy and E-Epilepsy Inclusion); however, both had lower installation rates (5000+ and 100+, respectively). Apps available only in Google Play or both Google Play and App Store had higher installation rates than the those available in App Store only (n=6), which include Seizure First Aide, E-Epilepsy Inclusion, ElFY Epilepsy, Epi & Me 2, M.E., and SAMi3 Sleep Activity Monitor (Tables 2 and 3).

**Table 3:** Name of the apps identified in this study and their eSM features (n=22).

App name (developer)	Operating system	Self-management features
Epilepsy Journal (Olly Tree Application)	Android	Helps to track seizure frequency; identify the effectiveness of epilepsy treatments; and input data on symptoms related to epilepsy that help HCPs to adjust medication dose or change the medication
Epilepsy Tool Kit (Epilepsy Society)	Both	Uses a new and interactive way by which it helps PWEs to manage their epilepsy
EpiDiary (EpiDiary Irody, Inc.)	Android	Keeps track of seizures, medicines, sleep, and how one feels daily
Young Epilepsy (Synergix Health Ltd.)	Both	Useful for young people with epilepsy and for parents or caregivers of a child who has epilepsy. Provides up-to-date information and has portal, video, and diary functionalities that help track seizures and manage symptoms
Seizure Log (Seizure Tracker LLC)	Both	Records seizure attacks
Simple Seizure (NA^a^)	Android	Used as a diary to manage epilepsy
My Seizure Diary (Epilepsy Foundation)	Both	Epilepsy management includes self-monitoring and tracking, managing medications, and communicating with health care providers
Epilepsy Ireland Diary App (DXC Technology)	Both	Used to track and record seizures and also determine trigger factors
Seizure First Aide (Afixia LLC)	iOS	Educates about epilepsy
Win Over Epilepsy (WOE) (Bee Mobile Pvt. Ltd.)	Android	Helps to keep track of seizures, medicines, visits, etc.
Birdhouse – for Epilepsy (Birdhouse LLC)	Both	Helps to identify seizure triggers, manage a medication log, evaluate diets, and ensure the path to success
EpApp (PENNSW & The Sydney Children’s Hospitals Network)	Both	Provides information about epilepsy; also has a tool that supports self-management for PWEs and their parents
BioMark Health Epilepsy (NA)	Android	Offers multiple functionalities (eg, passive data collection, medication reminders, quantified self-data collection, and care-team management) to understand epilepsy better and achieve more peace of mind for caregivers
E-Action Info (The ImageFactory)	Both	Teaches and increases the knowledge on epilepsy in a fun and easy way
E-Epilepsy Inclusion (The Hong Kong Society for Rehabilitation)	iOS	Assists in enhancing epilepsy self-management; helps to communicate with health care providers; and educates the public about epilepsy
ElFY Epilepsy (ELFY APP)	iOS	Tracks the right medication every day
Helpilepsy – Epilepsy Assistant & Diary (M4KEIT)	Both	Assists, tracks, and shares epilepsy records more easily and effectively
Epilepsy Help (Dr. Bindu Menon Foundations)	Android	Helps patients with epilepsy to organize and upload their medical data, keep medicine alarm and seizure alert, track appointment schedule, and know about epilepsy
Epilepsy Safe (Comalf)	Android	Helps to receive emergency aid from passersby in case of seizure attack in a public place
Epi & Me 2 (HandMe)	iOS	Assists in epilepsy management such as collecting and storing data on seizures, medication adherence, and life circumstances
M.E. (Epilepsy Foundation of New Jersey)	iOS	Useful for people with epilepsy and their families to manage their epilepsy
SAMi3 Sleep Activity Monitor (HiPass Design LIC)	iOS	Monitors the sleep activity of the patient for the caregiver and family members to carefully observe for any abnormal movement at night

^a^NA: not available.

### Mobile App Description

This section describes the 22 apps which have been identified through this systematic review. Table 3 presents the name of the app and the self-management features included, while the eSM domain(s) they cover are listed in Table 4.

**Table 4:** App names and domains of self-management.

App name	Treatment management	Medication adherence	Health care communication	Seizure tracking	Seizure response	Safety	Wellness	Social support	Coping	Stress management	Proactivity	Total domains
Epilepsy Journal	✓	✓	✓	✓								4
Epilepsy Tool Kit	✓	✓	✓	✓	✓	✓						6
My Seizure Diary	✓	✓	✓	✓	✓			✓				6
EpApp	✓		✓	✓	✓							4
Young Epilepsy	✓	✓	✓	✓	✓	✓	✓	✓	✓	✓		10
Helpilepsy – Epilepsy Assistant & Diary	✓	✓		✓	✓							4
EpiDiary	✓	✓	✓	✓								4
Simple Seizure			✓	✓	✓							3
Epilepsy Help	✓	✓		✓	✓	✓					✓	6
BioMark Health Epilepsy	✓	✓		✓	✓							4
Epilepsy Ireland Diary App			✓	✓	✓							3
Epilepsy Safe	✓	✓	✓	✓				✓				5
Win Over Epilepsy (WOE)	✓	✓	✓	✓	✓			✓				6
Birdhouse – for Epilepsy	✓	✓	✓	✓	✓	✓						6
E-Action Info	✓	✓	✓	✓	✓	✓						6
E-Epilepsy Inclusion	✓	✓	✓	✓	✓	✓			✓	✓	✓	9
ElFY Epilepsy	✓	✓	✓	✓	✓		✓					6
Seizure Log	✓		✓	✓	✓							4
Epi & Me 2	✓	✓		✓	✓							4
Seizure First Aide				✓	✓	✓						3
M.E.				✓	✓							2
SAMi3 Sleep Activity Monitor				✓	✓	✓						3

### Treatment Management

A total of 17/22 apps (77%; eg, Epilepsy Journal, Epilepsy Tool Kit, Young Epilepsy, Epilepsy Help, Epi & Me 2) help patients self-manage their treatment. The common features in these apps were a reminder for treatment, appointment, and recommendations and advice of HCPs that should be followed.

### Medication Adherence

A total of 15 apps, such as the Epilepsy Journal, My Seizure Diary, Birdhouse – for Epilepsy, EpiDiary, BioMark Health Epilepsy, Win Over Epilepsy (WOE), ElFY Epilepsy, and Seizure Log, were found to have features related to medication adherence. These apps enabled PWEs to establish and record a medication list, including the number of repeats remaining, the personalized dose, how many days the supply would last, and an alert reminder. Moreover, they provide PWEs a chance to register their time of medication, type of medication received, and frequency of acquiring the medication with their medication photo attached.

### Health Care Communication

The success of the patient-centered model depends on communication between HCPs and patients [20], which strengthens their relationship and facilitates better treatment. In our study, 16 apps (73%), such as E-Epilepsy Inclusion, Simple Seizure Diary, Epilepsy Tool Kit, and Epi & Me, covered health care communication. These apps allow the PWEs to send their reports to their HCPs through email or share it as an efile during an appointment. This feature helps patients, caregivers, and HCPs to establish a relationship, which is extremely important. In addition, some apps allow the patient and HCPs to monitor the side effects of epilepsy, such as depression, anxiety, unstable emotions, and sleep problems.

### Seizure Tracking

Seizure tracking provides critical information that helps the health care professional to evaluate the medication and health treatment received by the patients. All the apps cover seizure tracking and provide the features of tracking, such as registration of seizure time, duration, type and frequency of seizure, seizure triggers, and video recordings of the seizure.

### Seizure Response

In our study, 19 apps (86%), such as Young Epilepsy, Seizure First Aide, Help Epilepsy, Seizure Log, BioMark Health Epilepsy, Epilepsy Tool Kit, Epilepsy Ireland Diary App, and Epilepsy Safe, cover the domain of seizure response. These apps include features such as first-aid instructions on how one can help someone when they have a seizure and when to dial 999. A customized emergency SMS text message can be sent with a touch of a button during the aura stage or following a seizure. The user can automatically send an alert message (with GPS location) to his/her family through the Epilepsy Safe app.

### Safety

Eight apps (36%), such as Birdhouse – for Epilepsy and E-Action Info, covered the safety domain, which relates to avoiding risks that are detrimental to PWEs. One app from the App Store provided information for patients regarding the course of action during a seizure attack.

### Wellness

Three apps, namely, ElFY Epilepsy, Birdhouse – for Epilepsy, and Young Journal, covered the wellness domain (compatible with both iOS and Android) and provide the PWEs with some type of wellness-related information, such as diet and exercise. The Birdhouse – for Epilepsy app also tracks food consumption to help identify associations with seizure activity.

### Social Support

Four apps, namely, My Seizure Diary, Young Epilepsy, Epilepsy Safe, and Win Over Epilepsy, covered the social support domain and contained some stories related to epilepsy. This domain includes sharing stories or experiences with PWEs and getting some advice regarding epilepsy.

### Coping and Stress Management

Coping, stress management, and proactivity are less popular domains and are included in only 2 apps, namely, ElFY Epilepsy and Young Epilepsy. These cover topics on managing stress; doing something that reduces sadness; and encourage PWEs to continue hobbies, relaxation, or exercises that help prevent seizure.

### Proactivity

Only 2 apps provided some information related to proactivity (Epilepsy Help and E-Epilepsy Inclusion), and included some advice that may avoid situations or things that might cause a seizure.

## Discussion

### Principal Findings

In this review, 22 SM apps for epilepsy were found. The apps with the highest installation frequency were Epilepsy Journal, Epilepsy Tool Kit, and EpiDiary (10,000+ for each), and these cover the 4 most important domains of eSM, namely, treatment management, medication adherence, health care communication, and seizure tracking. However, as nearly 50 million people are estimated to have epilepsy worldwide [1,2], these apps are underutilized.

Monitoring seizure episodes assists in increasing the possibility of capturing seizures and tracking the evolution of the disease [21]. Using paper diaries to collect data related to patient’s symptoms is a challenging task. Thus, mobile apps have become a great tool that help PWEs to cope with and control their condition. These apps also aid PWEs to record their data and evaluate the effect of medication and treatment administered in consultation with their HCPs. A well-designed app provides a good opportunity for both PWEs and HCPs to manage epilepsy and select the best option together.

Apps with the highest download/installation frequency reflect increased acceptability by users, despite covering less domains. Most of these apps focus on seizure tracking because this provides valuable information that helps in the management of the disease [21]. A high percentage (60%-70%) of patients can become seizure free when they promptly take the effective medication. Adherence is defined by the World Health Organization as “the extent to which a person’s behavior—taking medication, following a diet, and executing lifestyle changes—corresponds with the agreed recommendations from a HCP” [22].

However, finding an appropriate antiepileptic drug (AED) can be a long and challenging process because patients struggle to report their seizure frequency [23]. Providing seizure information remains critical to help neurologists in selecting the correct dose of AEDs for their patients; efficiency of AED in long-term treatments should also be evaluated [23].

Nonadherence is a critical issue for PWEs [24] and can lead to an increase in seizure frequency [25]. PWEs generally do not adhere to their medication, which negatively affects their situation [26]. Nonadherence to medications usually decreases the quality of treatment outcomes, maximizes the consultations and hospitalization, and increases the health care cost [27]. Despite the risks associated with not taking medications, 50% of the patients with a chronic condition fail to adhere to their treatment recommendations [22]. Adherence requires active involvement of the patient and a therapeutic alliance between the patient and his/her physician [28]. Therefore, most apps have features to support medication management.

According to our review, most of the apps cover at least one or more domains of eSM, as recommended by the World Health Organization [4]. Each app has features that allow PWEs to self-manage epilepsy. However, not all the apps analyzed covered all 11 domains. Every app focused on different domains of eSM. The Young Epilepsy app is the only one that covered most (10 overall) eSM domains (user rating: 4.4), whereas the My Seizure Diary app covered only 6 domains and had the lowest user rating (2.2).

Without effective management, PWEs will have poor quality of life, and so mHealth apps are considered promising tools that will assist and fill the gap in eSM. End users should be part of design and development stages of apps to increase the quality of apps and achieve the app’s intended goal(s).

### Study Limitations

We only included English apps because this study aimed to review the current epilepsy apps from an international perspective, and not just from a Malaysian perspective. However, the apps were accessed through the Malaysian App Store and Google Play sites as stated in the “Methods” section. The research was conducted in Malaysia, and the apps that are available for iOS and Android in this country differ from those in the United States, while some are not available in our country, which possibly affects the overall analysis. The study included only free apps. Some with reasonable prices might be added to make the study more valuable. Many apps were excluded from the study because their languages were not English, although some of these have great features. Only Android- and iOS-compatible apps were targeted in this research. However, many related apps could be available for other mobile operating systems, such as BlackBerry or LiteOS (Huawei).

### Conclusion and Future Work

A total of 22 apps were found to support different eSM domains; however, almost all of these were underutilized (ie, had less installation rate). Thus, to improve the efficacy and availability of these apps, we propose the following: (1) involve various stakeholders, such as physicians, pharmacists, and PWEs, during the development of mHealth apps; (2) assess the efficacy and acceptance of these apps objectively by performing a usability analysis; and (3) promote the apps so that they benefit more PWEs. Malaysia is a multiracial country which is dominated by 3 racial groups, namely, Malay, Indian, and Chinese. In the future, we will also analyze similar apps in local languages to ensure all important features are considered.

## Abbreviations

eSM: epilepsy self-management
HCP: health care provider
PWEs: patients with epilepsy
SM: self-management
